# Understanding the Perspectives of Hail City Population on the Confidentiality and Privacy of Digital Health and Medical Information

**DOI:** 10.7759/cureus.63752

**Published:** 2024-07-03

**Authors:** Anas Alhur

**Affiliations:** 1 Health Informatics, University of Hail College of Public Health and Health Informatics, Hail, SAU

**Keywords:** saudi arabia, hail city, demographic factors, health information safety, confidentiality, privacy, digital health

## Abstract

Background

In the era of rapid digital advancement, the confidentiality and privacy of digital health and medical data have become paramount concerns. This study investigates the perspectives of individuals residing in Hail City regarding these critical issues, with a particular emphasis on the influence of demographic factors such as age, gender, and computer proficiency on individuals’ discomfort with health professionals using computers and their trust in researchers. Gaining a deeper understanding of these factors is vital for the development of targeted interventions aimed at enhancing patient comfort and trust in digital health/medical technologies.

Methodology

This study employed a descriptive cross-sectional design, involving a survey of 775 individuals aged 18 and above in Hail City. The questionnaire was designed to gather information on participants’ demographic characteristics, computer proficiency, experiences with digital health and medical information, and perceptions of health information safety and privacy. To examine the associations and predictive relationships between variables, chi-square tests, correlation analyses, and logistic regression were performed.

Results

Significant associations were found between gender and discomfort with health professionals using computers (chi-square = 60.29, p < 0.0001), and between age and trust in researchers regarding the privacy of medical information (chi-square = 50.14, p < 0.0001). Positive correlations were observed between computer proficiency and perception of health information safety (r = 0.12, p = 0.0002), while a negative correlation was found between computer ownership and avoidance of medical tests due to privacy concerns (r = -0.08, p = 0.03). Logistic regression analysis identified age, gender, and computer proficiency as significant predictors of discomfort with health professionals using computers. The findings highlight the crucial role that demographic factors play in shaping attitudes toward the privacy and security of digital health and medical information.

Conclusions

The findings of this study highlight the crucial role that demographic factors play in shaping attitudes toward the privacy and security of digital health and medical information. Gender and age were found to significantly influence individuals’ levels of discomfort and trust, while computer proficiency was shown to enhance perceptions of safety. Based on these findings, the researchers recommend implementing targeted interventions, such as gender-sensitive training programs and initiatives, to enhance digital literacy and improve patient comfort and trust in digital health technologies.

## Introduction

In the digital age, the confidentiality and privacy of health and medical data have become increasingly critical. With the widespread adoption of electronic health records (EHRs) and other digital health technologies, ensuring the security of personal health information is paramount. The importance of safeguarding digital health and medical information cannot be overstated. The rapid growth in EHR adoption brings both opportunities and challenges in terms of information security [1]. Maintaining the confidentiality of health information is crucial for fostering trust among patients and healthcare providers.

Previous research has highlighted varying levels of concern regarding digital health and medical information privacy. Trust in healthcare providers significantly impacts individuals’ willingness to share personal health and medical information electronically [2]. A survey by the Pew Research Center revealed that a substantial portion of the population is concerned about the security of their EHRs, with 55% of respondents expressing worry about unauthorized access to their health information [3].

In Saudi Arabia, there has been a growing emphasis on improving healthcare services through digital transformation. However, addressing privacy concerns is essential for successful digital health adoption [4]. A recent study in Saudi Arabia found that 43.5% of participants reported average computer skills, and 8.9% expressed discomfort with healthcare professionals using computers [5-7].

This study aims to contribute to the existing body of knowledge by examining the influence of demographic factors on individuals’ attitudes and perceptions toward digital health information privacy and security in Hail City. By understanding these factors, targeted interventions can be developed to enhance patient comfort and trust in digital health and medical technologies, ultimately leading to better engagement with digital health services and improved health outcomes.

Objectives

This study aims to describe the demographic characteristics, technology usage, and computer proficiency among respondents in Hail City, Saudi Arabia. It examines the privacy concerns and perceptions regarding health and medical information safety, trust, and access to health and medical information among respondents in Hail City, Saudi Arabia. Further, it aims to investigate the associations and correlations between selected variable pairs, such as gender and discomfort with health professionals using computers, age and trust in researchers regarding medical information privacy, computer proficiency and perception of health and medical information safety, and ownership of a computer and avoidance of medical tests due to privacy concerns using appropriate statistical tests (e.g., chi-square tests for independence and correlation analysis). Finally, it aims to identify significant predictors of discomfort with health professionals using computers among respondents in Hail City, Saudi Arabia, using logistic regression analysis, considering demographic factors such as age, gender, and computer proficiency, and determining their associated odds ratios and confidence intervals.

## Materials and methods

Study design

This cross-sectional descriptive study aimed to investigate the perspectives of individuals in Hail City, Saudi Arabia, on the confidentiality and privacy of their digital health and medical data. The study focused on examining the influence of demographic factors, such as age, gender, and computer proficiency, on individuals’ discomfort with health professionals using computers and their trust in researchers.

Study setting and population

The study was conducted in Hail City, a major urban center in the northern region of Saudi Arabia. The target population consisted of the population of Hail City aged 18 years and above. To ensure a representative sample, participants were selected to reflect the diverse demographic characteristics of the city, including various age groups, genders, educational backgrounds, and levels of computer proficiency.

Sample size and sampling technique

The study surveyed a total of 775 participants, calculated using Cochran’s formula for estimating a population proportion, with a confidence interval of 95% and a margin of error of 5%. The assumed proportion was 0.5, which maximizes the sample size for a given confidence level and margin of error. A stratified random sampling technique was employed to ensure representativeness. The population was divided into strata based on key demographic variables, including age, gender, and educational level. Random samples were then drawn from each stratum in proportion to their representation in the population.

Data collection instrument

A structured questionnaire, specifically designed for this study, was used to collect data from the participants. The detailed questionnaires are provided as supplementary materials. Appendix 1 includes Questionnaire A, which covers demographic information, computer proficiency, and experiences with digital health and medical information. Appendix 2 presents Questionnaire B, which addresses perceptions of health information safety, privacy concerns, and trust in health professionals and researchers.

The questionnaire consisted of the following four main sections: (1) Demographic information: This section gathered data on participants’ age, gender, educational level, and health status. (2) Computer proficiency: Participants’ self-reported computer proficiency levels were assessed in this section. (3) Experiences with digital health information: This section included questions regarding computer ownership, experiences with serious breaches of health information, and avoidance of medical tests due to privacy concerns. (4) Perceptions of health and medical safety and privacy: Participants’ comfort with health professionals using computers, trust in researchers regarding medical information privacy, and awareness of rights to access and modify medical records were evaluated in this section.

Questionnaire development and validation

The questionnaire was developed through a comprehensive review of existing literature and validated scales relevant to the study’s objectives. It was pretested on a sample of 30 participants, who were not included in the main study. The pretest participants reflected the demographics of the main study population, including a mix of ages, genders, and educational levels.

Based on the feedback from the pretest, necessary modifications were made to improve clarity and relevance. This included rewording ambiguous questions, adding additional options to better capture respondents’ views, and ensuring the overall coherence of the questionnaire. The pretest was conducted one month before the main data collection period, allowing sufficient time for refinement based on the feedback received.

Data collection procedure

The data collection process spanned a period of three months. Participants were recruited from various public settings, including shopping malls, healthcare centers, and universities in Hail City. Before administering the questionnaire, the researchers provided the participants with a concise explanation of the study’s purpose and reassured them of the confidentiality of their responses. Informed consent was obtained from all participants before they completed the questionnaire.

Data analysis

The collected data were coded and entered into SPSS version 26 (IBM Corp., Armonk, NY, USA) for comprehensive analysis. The data analysis plan encompassed both descriptive and inferential statistical techniques.

Descriptive Statistics

Frequencies and percentages were computed for categorical variables, while means and standard deviations were calculated for continuous variables to summarize the data.

Chi-Square Tests for Independence

To investigate the associations between categorical variables, specifically gender and discomfort with health professionals using computers, and age and trust in researchers regarding medical information privacy, chi-square tests were conducted.

Correlation Analysis

Pearson correlation coefficients were calculated to assess the relationships between computer proficiency and perception of health information safety, as well as between computer ownership and avoidance of medical tests due to privacy concerns.

Logistic Regression

To identify significant predictors of discomfort with health professionals using computers, a logistic regression analysis was performed. The independent variables included in the model were age, gender, and computer proficiency.

Ethical considerations

This study was conducted in accordance with the ethical guidelines and principles set forth by the Ethical Approval Committee from the Research Department at Hail Health Cluster. The study received ethical approval from the committee (approval number: 2024-123). The Ethical Approval Committee thoroughly reviewed the study protocol, questionnaire, and informed consent procedures to ensure that the research adhered to the highest standards of ethical conduct. The committee assessed the study’s compliance with the Declaration of Helsinki and relevant national and institutional regulations governing human subject research.

## Results

The study included 775 individuals from Hail City, with 61.43% (476) females and 38.57% (299) males. Most respondents were aged 26-33 years (62.66%, 478), followed by 18-25 years (22.41%, 171). The 34-44 years group comprised 9.17% (70), and those aged 45+ were 5.76% (44). Regarding education, 77.81% (610) held a Bachelor’s degree, while 21.81% (171) had completed high school. Regarding health status, 50.06% (389) reported their health as very good, 38.22% (297) as good, 10.94% (85) as moderate, and 0.77% (6) as bad (Table 1).

**Table 1:** Demographic characteristics of the study population in Hail City.

Category	Frequency (n)	Percentage (%)
Gender		
Female	476	61.43
Male	299	38.57
Age (years)		
26–33	478	62.66
18–25	171	22.41
34–44	70	9.17
45+	44	5.76
Education status		
Bachelor	610	77.81
High school	171	21.81
Health status		
Very good	389	50.06
Good	297	38.22
Moderate	85	10.94
Bad	6	0.77

A substantial proportion of the study population had access to personal computers, with 77.19% (600 individuals) reporting computer ownership. This high level of computer ownership was reflected by a mean of 0.772 and a standard deviation of 0.420, indicating widespread adoption of technology among respondents.

Regarding computer proficiency, 61.37% (483 individuals) rated their proficiency as above average, suggesting a significant portion of the population possessed advanced computer skills. The remaining 38.63% (304 individuals) considered their proficiency to be average, indicating a solid foundation of computer literacy. The mean proficiency score was 0.807, with a standard deviation of 0.244, reinforcing the notion that respondents in Hail City possess relatively high computer skills (Table 2).

**Table 2:** Technology usage and computer proficiency among respondents in Hail City.

Category	Frequency (n)	Percentage (%)	Mean	Standard deviation
Owns a computer	600	77.19	0.772	0.42
Does not own a computer	177	22.81		
Above average proficiency	483	61.37	0.807	0.244
Average proficiency	304	38.63		

The majority of respondents (94.29%, 660 individuals) reported not experiencing a serious breach of their health information, while 5.71% (40 individuals) indicated that they had experienced such a breach. The mean score for experiencing a serious breach was 0.057, with a standard deviation of 0.232, suggesting that breaches were relatively uncommon.

Regarding avoidance of medical tests due to privacy concerns, most respondents (88.99%, 696 individuals) did not avoid tests, whereas 11.01% (86 individuals) did. The mean score was 0.110, with a standard deviation of 0.313, indicating a moderate impact of privacy concerns on medical testing behavior.

When asked about requests to doctors not to record or modify health issues in medical records, 93.06% (695 individuals) did not make such requests, while 6.94% (52 individuals) did. The mean score was 0.070, with a standard deviation of 0.255, showing that this behavior was rare among respondents.

Discomfort with health professionals using computers was reported as not an issue by 63.85% (498 individuals), while 11.28% (88 individuals) expressed discomfort, and 24.87% (194 individuals) were uncertain or maybe uncomfortable. The mean score was 0.184, with a standard deviation of 0.330, reflecting varied feelings toward computer use in healthcare settings.

Perception of health information safety showed that 63.79% (502 individuals) felt their health information was safe, 9.10% (71 individuals) did not feel safe, and 26.58% (207 individuals) were unsure. The mean perception score was 0.776, with a standard deviation of 0.328, indicating a generally positive perception of health information safety.

Concern about violation of personal health information was low, with 76.81% (573 individuals) not concerned, while 23.19% (173 individuals) being concerned. The mean score was 0.184, with a standard deviation of 0.330, showing that concerns about violations were not widespread.

When considering the biggest threat to medical record privacy, 59.24% (461 individuals) perceived both internal and external threats equally, while 40.76% (317 individuals) perceived neither as a significant threat. The mean score was 0.517, with a standard deviation of 0.500, indicating a balanced perception of privacy threats (Table 3).

**Table 3:** Privacy concerns and perceptions regarding health Information safety among respondents in Hail City.

Category	Frequency (n)	Percentage (%)	Mean	Standard deviation
No breach experienced	660	94.29	0.057	0.232
Breach experienced	40	5.71		
No medical test avoidance	696	88.99	0.11	0.313
Medical test avoidance	86	11.01		
No request for record modification	695	93.06	0.07	0.255
Request for record modification	52	6.94		
No discomfort with computers	498	63.85	0.184	0.33
Discomfort with computers	88	11.28		
Maybe discomfort	194	24.87		
Perception of safety	502	63.79	0.776	0.328
No perception of safety	71	9.1		
Maybe perception of safety	207	26.58		
No concern about violation	573	76.81	0.184	0.33
Concern about violation	173	23.19		
Both internal and external threats	461	59.24	0.517	0.5
Neither threat	317	40.76		

The findings reveal that respondents placed varying levels of trust in different entities regarding the privacy of their medical information. Nurses and other hospital staff were the most trusted, with 53.75% (423 individuals) expressing confidence in these healthcare professionals. Doctors followed closely, with 38.50% (303 individuals) trusting them with their medical information privacy. Pharmacists were trusted by a smaller proportion of respondents at 7.75% (61 individuals). The mean trust score for these entities was 0.057, with a standard deviation of 0.232, indicating a diverse range of trust levels among the study population.

When it came to trusting researchers with medical information privacy, respondents demonstrated a higher level of confidence in government health researchers compared to university-based health researchers. A substantial 61.05% (472 individuals) trusted government health researchers, while 38.95% (301 individuals) placed their trust in university-based health researchers. The mean trust score for researchers was 0.829, with a standard deviation of 0.237, reflecting a generally high level of trust in these professionals.

The respondents’ understanding of their right to access and modify their medical records in Saudi Arabia was mixed. Nearly half of the respondents (49.42%, 256 individuals) believed they did not have this right, while approximately one-third (32.63%, 169 individuals) were confident they possessed this right. The remaining 17.95% (93 individuals) expressed uncertainty about their rights. The mean score for this perception was 0.498, with a standard deviation of 0.500, highlighting the varied levels of understanding among the population concerning their rights to access and modify their medical records.

Regarding the awareness of laws preventing the use of medical data without consent in Saudi Arabia, respondents demonstrated a moderate level of knowledge. Over half of the respondents (55.40%, 436 individuals) were aware of such laws, while 21.35% (168 individuals) were unaware, and 21.60% (170 individuals) were uncertain. The mean awareness score was 0.673, with a standard deviation of 0.407, indicating that while a majority of respondents were informed about legal protections for medical data privacy, there remains room for improvement in raising awareness among the population (Table 4).

**Table 4:** Trust and access to health information among respondents in Hail City.

Category	Frequency/Percentage (%)
Entities trusted with medical information privacy	0.057/0.232
Nurses and other hospital staff	423/53.75%
Your doctor	303/38.50%
Pharmacist	61/7.75%
Trust in researchers regarding medical information privacy	0.829/0.237
Government health researchers	472/61.05%
Health researchers at universities	301/38.95%
Right to access and modify medical records in Saudi Arabia	0.498/0.500
No	256/49.42%
Yes	169/32.63%
Maybe	93/17.95%
Laws preventing use of medical data without consent in Saudi Arabia	0.673/0.407
Yes	436/55.40%
No	168/21.35%
Maybe	170/21.60%

The chi-square test results indicated significant associations between the specified variable pairs. For gender and discomfort with health professionals using computers, the chi-square statistic was 60.29, with a p-value of less than 0.0001, suggesting a significant association. This implies that gender significantly influences discomfort with health professionals using computers.

Similarly, the chi-square statistic for age and trust in researchers regarding medical information privacy was 50.14, with a p-value of less than 0.0001, indicating a significant association. This suggests that age significantly influences trust in researchers regarding medical information privacy (Table 5).

**Table 5:** Chi-square test results for independence. This table displays the results of the Chi-Square tests for independence between key variable pairs. The table includes the Chi-Square statistic, p-value, and conclusion for each pair. Significant associations indicate that the variables are not independent of each other and highlight important relationships. Specifically, the table shows a significant association between gender and discomfort with health professionals using computers, as well as between age and trust in researchers regarding the privacy of medical information.

Variable pair	Chi-square statistic (χ²)	P-value	Conclusion
Gender and discomfort with health professionals using computers	60.29	<0.0001	Significant association
Age and trust in researchers regarding medical information privacy	50.14	<0.0001	Significant association

The correlation analysis showed a significant positive correlation (r = 0.12, p = 0.0002) between computer proficiency and the perception of health information safety. This indicates that as computer proficiency increases, the perception of health information safety also tends to increase. Additionally, there was a significant negative correlation (r = -0.08, p = 0.03) between owning a computer and avoiding medical tests due to privacy concerns. This suggests that individuals who own computers are less likely to avoid medical tests due to privacy concerns (Table 6).

**Table 6:** Correlation analysis results. This table presents the results of the correlation analysis between key variables. The table shows the correlation coefficients (r) and p-values for the relationships between computer proficiency and perception of health information safety, as well as ownership of computers and avoidance of medical tests due to privacy concerns. Significant correlations indicate how these factors are associated with individuals' perceptions and behaviors related to health information safety and privacy.

Variable pair	Correlation coefficient (r)	P-value	Interpretation
Computer proficiency and perception of health information safety	0.12	0.0002	Significant positive correlation. As computer proficiency increases, perception of health information safety also increases
Ownership of computer and avoidance of medical tests due to privacy concerns	-0.08	0.03	Significant negative correlation. Individuals who own computers are less likely to avoid medical tests due to privacy concerns

For each one-year increase in age, the log odds of experiencing discomfort with health professionals using computers increased by 0.021. This result was statistically significant (p = 0.036), indicating that older individuals were slightly more likely to experience discomfort. Males were significantly more likely to experience discomfort with health professionals using computers compared to females, with an estimate of 1.462 (p < 0.0001). Higher computer proficiency was associated with a lower likelihood of experiencing discomfort with health professionals using computers, with an estimate of -0.736 (p = 0.010) (Table 7).

**Table 7:** Logistic regression summary. This table presents the logistic regression analysis results for predicting discomfort with health professionals using computers. The table includes the estimates of the coefficients, standard errors, z-values, p-values, and 95% confidence intervals (CIs) for each predictor variable. The predictors are age, gender (with males coded as 1 and females as 0), and computer proficiency. Significant predictors are identified based on their p-values, highlighting the factors that significantly influence the likelihood of experiencing discomfort with health professionals using computers.

Variable	Estimate	Standard error	z-value	P-value	95% CI (lower)	95% CI (upper)
Constant	-3.256	0.783	-4.157	<0.0001	-4.79	-1.721
Age	0.021	0.01	2.1	0.036	0.001	0.041
Gender (male = 1, female = 0)	1.462	0.312	4.684	<0.0001	0.85	2.073
Computer proficiency	-0.736	0.285	-2.579	0.01	-1.295	-0.177

## Discussion

This study sought to investigate the perspectives of Hail City residents on the confidentiality and privacy of their digital health and medical data, with a particular emphasis on how demographic factors influence discomfort with health professionals using computers and trust in researchers. The significant associations discovered in this study offer valuable insights into the interplay between demographic variables and attitudes toward digital health information security.

The noteworthy association between gender and discomfort with health professionals using computers highlights a critical area for targeted interventions. Previous research has consistently demonstrated that gender can shape attitudes toward technology. For example, studies have found that men tend to experience higher levels of discomfort and anxiety when using digital health tools compared to women. This difference may be attributed to variations in technology use patterns, with women generally being more engaged in health-related technology use. Addressing this discomfort through gender-sensitive training programs could enhance the user experience for men and increase their comfort with digital health tools [8].

The relationship between age and trust in researchers regarding medical information privacy highlights the importance of considering age-related differences in digital literacy and trust. Older adults often display lower levels of trust in digital health systems due to concerns about privacy and past experiences with technology breaches. Enhancing digital literacy among older adults through targeted educational programs could alleviate these concerns and improve their trust in digital health systems. Furthermore, designing user interfaces that are more accessible and user-friendly for older populations can further enhance their trust and engagement [9,10].

The positive correlation between computer proficiency and perception of health information safety suggests that improving digital skills can boost individuals’ confidence in the security of their health information. This finding is consistent with existing literature indicating that higher digital literacy is associated with greater confidence in using digital health technologies [11-13]. Healthcare providers should consider implementing programs that enhance patients’ computer proficiency, thereby potentially reducing privacy concerns and increasing engagement with digital health services [14].

The negative correlation between computer ownership and avoidance of medical tests due to privacy concerns suggests that familiarity with technology reduces privacy-related apprehensions. This aligns with findings that increased exposure to and familiarity with digital technologies can alleviate privacy concerns [2,15-17]. Encouraging technology use and familiarity through community programs could reduce privacy-related barriers to healthcare access [17-22].

Implications for practice and policy

These findings have significant implications for healthcare practice and policy. Healthcare providers should develop and implement training programs tailored to different demographic groups to address specific discomforts and enhance trust in digital health tools. Policymakers should consider these demographic factors when designing and promoting digital health initiatives to ensure they are inclusive and effectively address the needs of diverse populations.

Moreover, the study emphasizes the importance of fostering digital literacy across all age groups. Programs aimed at improving digital skills could not only enhance the perception of health and medical information safety but also increase overall engagement with digital health services, and health informatics professionals can play a vital role here. This, in turn, can lead to better health outcomes as patients become more proactive in managing their health through digital platforms.

Limitations and future research

While this study provides important results, it is not without limitations. The cross-sectional design limits the ability to draw causal inferences. Longitudinal studies are needed to understand how perceptions and behaviors evolve over time. Additionally, the study focused on a specific geographic region, which may limit the generalizability of the findings. More studies should assess this research area nationwide and even internationally.

## Conclusions

In this study of Hail City individuals’ perspectives on the confidentiality and privacy of their digital health and medical information, the findings highlight the significant influence of demographic factors such as age, gender, and computer proficiency on attitudes toward digital health and medical information privacy and security. The results reveal that gender and age significantly impact discomfort with health professionals using computers and trust in researchers regarding medical information privacy, respectively, while computer proficiency positively correlates with the perception of health information safety, and computer ownership negatively correlates with the avoidance of medical tests due to privacy concerns. The results of this study carry significant weight for both healthcare practitioners and policymakers worldwide, mainly in Saudia Arabia. They demonstrate the necessity of developing and implementing customized interventions and educational initiatives that not only bridge the gender gap but also aim to enhance digital literacy among all age groups. By addressing these critical aspects, we can create an environment that nurtures patient comfort, builds trust, and encourages active engagement with digital health and medical technologies. Ultimately, this approach has the potential to pave the way for improved health outcomes by empowering patients to take a more proactive role in managing their well-being through the use of digital tools and platforms.

## Figures and Tables

**Figure 1:**
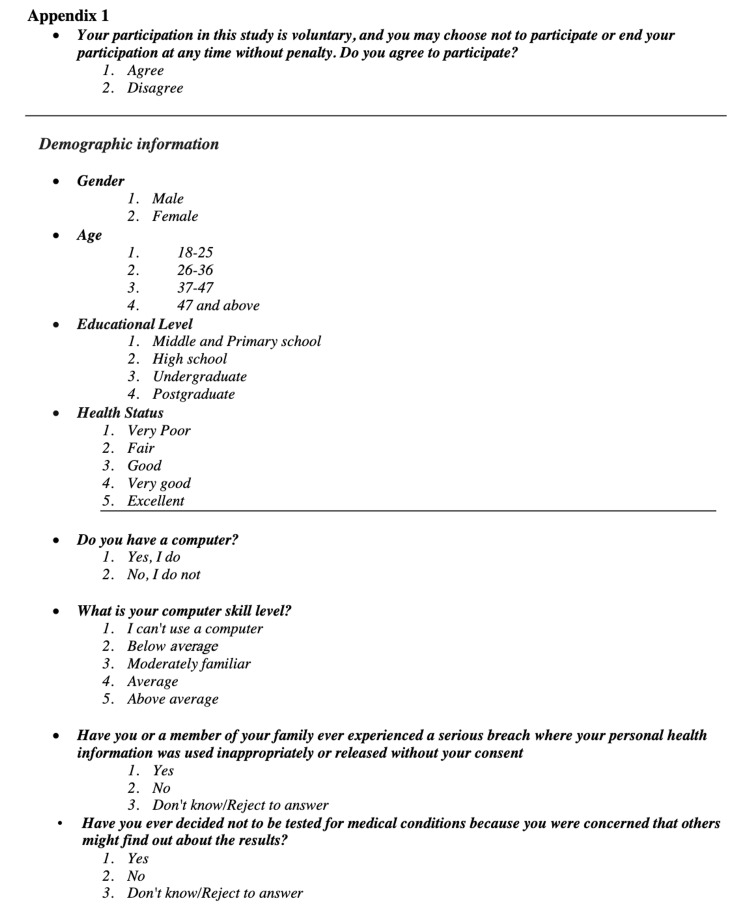
Survey questionnaire A on digital health and medical data confidentiality and privacy.

**Figure 2:**
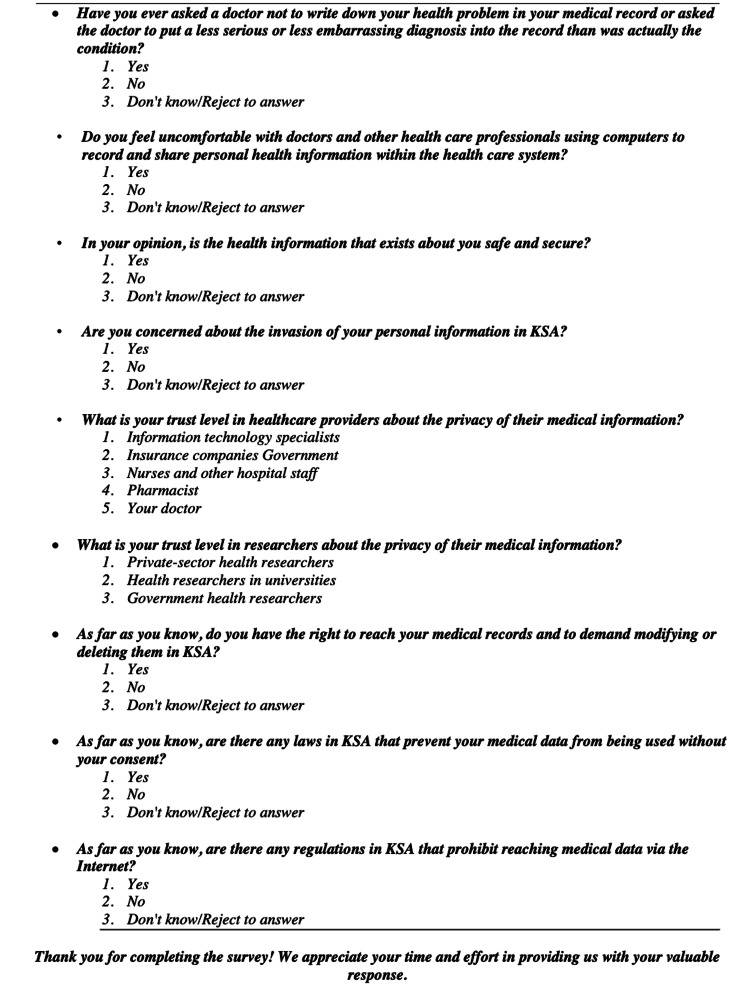
Survey questionnaire B on digital health and medical data confidentiality and privacy.

## Appendices

 Appendix 1

 Appendix 2
